# Olfactory perception and behavioral effects of sex pheromone gland components in *Helicoverpa armigera* and *Helicoverpa assulta*

**DOI:** 10.1038/srep22998

**Published:** 2016-03-15

**Authors:** Meng Xu, Hao Guo, Chao Hou, Han Wu, Ling-Qiao Huang, Chen-Zhu Wang

**Affiliations:** 1 State Key Laboratory of Integrated Management of Pest Insects and Rodents, Institute of Zoology, Chinese Academy of Sciences, Beijing 100101, P.R. China

## Abstract

Two sympatric species *Helicoverpa armigera* and *Helicoverpa assulta* use (Z)-11-hexadecenal and (Z)-9-hexadecenal as sex pheromone components in reverse ratio. They also share several other pheromone gland components (PGCs). We present a comparative study on the olfactory coding mechanism and behavioral effects of these additional PGCs in pheromone communication of the two species using single sensillum recording, *in situ* hybridization, calcium imaging, and wind tunnel. We classify antennal sensilla types A, B and C into A, B1, B2, C1, C2 and C3 based on the response profiles, and identify the glomeruli responsible for antagonist detection in both species. The abundance of these sensilla types when compared with the number of OSNs expressing each of six pheromone receptors suggests that HarmOR13 and HassOR13 are expressed in OSNs housed within A type sensilla, HarmOR14b within B and C type sensilla, while HassOR6 and HassOR16 within some of C type sensilla. We find that for *H. armigera,* (Z)-11-hexadecenol and (Z)-11-hexadecenyl acetate act as behavioral antagonists. For *H. assulta,* instead, (Z)-11-hexadecenyl acetate acts as an agonist, while (Z)-9-hexadecenol, (Z)-11-hexadecenol and (Z)-9-hexadecenyl acetate are antagonists. The results provide an overall picture of intra- and interspecific olfactory and behavioral responses to all PGCs in two sister species.

Despite more than 60 years of research into moth pheromone communication systems, crucial information regarding how segregation is achieved between closely related species and how sex pheromones might raise important barriers during speciation events is lacking. *Helicoverpa armigera* and *Helicoverpa assulta* both use (Z)-11-hexadecenal (Z11-16:Ald) and (Z)-9-hexadecenal (Z9-16:Ald) as their principal sex pheromone components but in almost opposite ratios, 98:2 and 5:95, respectively^1^,^2^,^3^. This fact renders this species pair highly interesting for comparative studies of mechanisms underlying detection and perception of pheromone blends. Besides the two aldehydes, other PGCs were also identified in female sex pheromone glands of *H. armigera*^2^,^3^,^4^,^5^ and *H. assulta*^1^,^3^, some of which play important roles in intra- and interspecific communication^1^,^2^,^6^,^7^. Table 1 lists the PGCs in *H. armigera* and *H. assulta* based on Wang *et al.*^3^ and summarizes their behavioral effects previously reported^1^,^2^,^4^,^6^,^7^,^8^,^9^. A comparative study on the functions of the PGCs in the two sympatric species would help to better understand intra- and interspecific communication of closely related species.

Like other Heliothine species, males of *H. armigera* and *H. assulta* take advantage of highly specialized olfactory sensory neurons (OSNs) housed in antennal sensilla to detect the PGCs^10^,^11^,^12^. The sensilla are classfied as A, B, and C types based on the response profiles of the housed OSNs to the pheromone components of Heliothine species^10^,^11^,^13^. Type A sensilla house an OSN responsive to the sex pheromone component Z11-16:Ald, type B one tuned to (Z)-9-tetradecenal (Z9-14:Ald). The response profiles of the type C sensilla are relatively broad and differ between species^10^,^11^. However, the responsiveness to one pheromone component and one interspecific signal compound is the common characteristic of type C sensilla. Matching the opposite ratios of the two components in pheromone blends of the two species, the type A sensilla are predominant in male *H. armigera*^8^, while the type C sensilla are predominant in male *H. assulta*^8^,^11^. The C type sensilla of *H. armigera* house neurons responding to Z9-16:Ald, Z9-14:Ald, (Z)-11-hexadecenol (Z11-16:OH) and (Z)-11-hexadecenyl acetate (Z11-16:Ac)^7^,^8^,^14^, while in *H. assulta* sensilla of the same type house OSNs responding to Z9-16:Ald, Z9-14:Ald and (Z)-9-hexadecenol (Z9-16:OH)^7^,^8^,^9^,^11^.

The response profiles of the OSNs are mainly determined by which odorant receptors (ORs) they express. ORs are seven transmembrane domain proteins and reside in the dendritic membranes of OSNs^15^,^16^,^17^. Unlike what is known in model organisms like *Drosophila*, little direct evidence has supported the attribution of ORs to OSNs in moth species. However, a group of ORs tuned to pheromone components and the related compounds, also called pheromone receptors (PRs) in the two species, have been deorphanized by measuring their response profiles to different PGCs when expressed in *Xenopus* oocytes^18^,^19^,^20^,^21^,^22^,^23^ or Sf9 cells^24^. HarmOR13 and HassOR13 are narrowly tuned to Z11-16:Ald^18^,^20^,^24^, while HarmOR14b and HassOR16 specifically respond to Z9-14:Ald^18^; HarmOR6 and HassOR6, instead, show a broader spectrum of responses, strong to Z9-16:OH, weaker to Z9-16:Ald, Z9-14:Ald and (Z)-9-hexadecenyl acetate (Z9-16:Ac)^18^. The correlation analysis between the population sizes of the different types of sensilla and the numbers of OSNs expressing the associated ORs could help assigning different ORs to OSNs and types of sensilla in the peripheral olfactory system.

The pheromonal information received by OSNs is conveyed to the primary brain center, the antennal lobe (AL). The AL structure of Heliothine species is sexually dimorphic, embodied by a macroglomerular complex (MGC). This structure is located at the input area of ALs of males and receives information from OSNs tuned to pheromone components and related compounds^25^,^26^,^27^,^28^. The MGC area in males of *Helicoverpa* species usually consists of three glomerular units, of which the largest, called cumulus, is innervated by the OSNs tuned to the major pheromone component. Of the other two glomeruli, named on the basis of their relative positions, one receives messages from secondary pheromone components, another takes other intra- or interspecies information^26^. Calcium imaging experiments showed that, in *H. armigera*, Z11-16:Ald elicited a robust response in the cumulus, while Z9-16:Ald activated the posterior dorsomedial unit (Dm-p)^8^. Z9-14:Ald elicited calcium activities in Dm-p and the anterior dorsomedial unit (DM-a)^7^. In *H. assulta,* the cumulus of the AL receives inputs from OSNs tuned to Z9-16:Ald, while the ventral unit is connected to OSNs tuned to Z11-16:Ald^8^,^11^. Staining of physiologically identified sensory neurons and projection neurons demonstrated that the dorsomedial unit (DM) in *H. assulta* processes interspecific information^11^,^13^,^29^. Calcium imaging confirmed that DM receives inputs from OSNs tuned to the antagonist Z9-14:Ald^7^. However, the response patterns in the MGC glomeruli after stimulation by other PGCs, Z9-16:OH, Z11-16:OH, Z9-16:Ac, Z11-16:Ac, have not been established in *H. armigera* and *H. assulta*.

In order to elucidate the role of the other PGCs in pheromone communication and interspecific behavioral isolation between *H. armigera* and *H. assulta*, and assign different PRs to OSNs and types of sensilla tuned to PGCs, we firstly performed a detailed classification of the types of antennal sensilla based on the response profiles of their housed OSNs to PGCs, and evaluated the population size of each type of sensilla in the two species; secondly we used *in situ* hybridization to determine the expression levels of six PRs and compared the values with the numbers of the associated OSNs in each species along the male antennae; thirdly we used *in vivo* optical imaging to study the transmission of peripheral input signals to antennal lobes in the brains of both species; finally we clarified the behavioral significance of PGCs in *H. armigera* and *H. assulta* with respect to pheromone communication and species isolation.

## Results

### Electrophysiological responses of OSNs in antennal sensilla responding to PGCs

**Figure 1:**
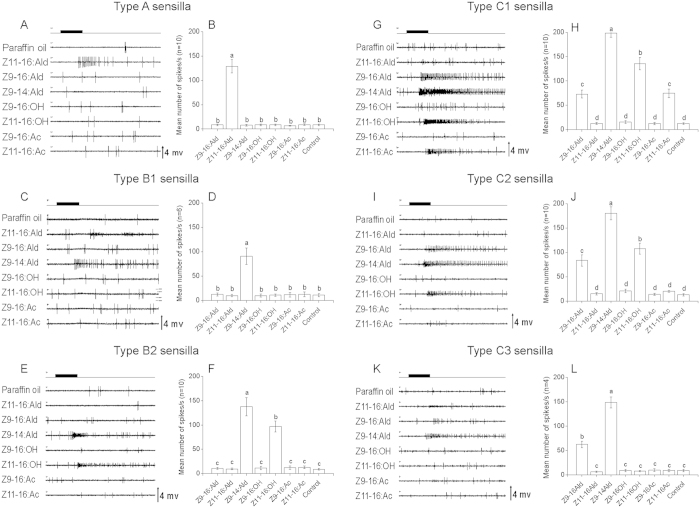
Response patterns of the associated olfactory sensory neurons housed in three types of sensilla in male antennae of *H. armigera*. For each type (or subtype) of sensilla, the left panels (**A**,**C**,**E**,**G**,**I**,**K**) show examples of the electrophysiological recordings. Black bars at the top of each panel show the duration of the stimuli. The right panels (**B**,**D**,**F**,**H**,**J**,**L**) report the spike frequencies (Mean ± SEM) which were calculated by counting the number of spikes during the first 200 ms of the response. In the C type sensilla, the total number of spikes from two co-localized neurons was counted. Columns with the same letters are not significantly different at *P* < 0.05. Paraffin oil was used as control.

**Figure 2:**
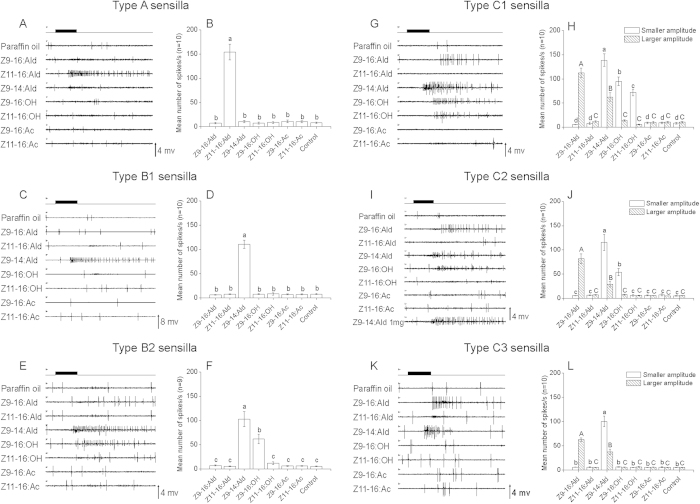
Response patterns of the associated olfactory sensory neurons housed in three types of sensilla in male antennae of *H. assulta*. For each type (or subtype) of sensilla, the left panels (**A**,**C**,**E**,**G**,**I**,**K**) show examples of the electrophysiological recordings. Black bars at the top of each panel show that duration of the stimuli. The right panels (**B**,**D**,**F**,**H**,**J**,**L**) report the spike frequencies (Mean ± SEM) which were calculated by counting the number of spikes during the first 200 ms of the response. Columns with the same letters are not significantly different at *P* < 0.05. Paraffin oil was used as control.

Based on the response profiles of their respective OSNs, the sensilla activated by PGCs in the 30-60 annuli of antennal flagella were first classified into A, B, C types consistently with previous reports^10^,^11^. The latter two types were further categorized into B1 and B2, and C1, C2, and C3, respectively (Figs 1 and 2).

The type A sensilla only responded to Z11-16:Ald (Figs 1A,B and 2A,B). The subtype B1 responded to Z9-14:Ald only, while subtype B2 responded to Z9-14:Ald as well as to an alcohol (Z11-16:OH in *H. armigera* and Z9-16:OH in *H. assulta*) (Figs 1C–F and 2C–F).

The type C sensilla showed common features in the two species, responding to both Z9-16:Ald and Z9-14:Ald (Figs 1G–L and 2G–L). Based on spike amplitudes, we could detect the presence of two OSNs in the type C sensilla of *H. assulta* (Fig. 2G–L). However, spikes with only one amplitude could be identified in C type sensilla of *H. armigera* (Fig. 2G–L), although our previous cross-adaptation test indicated the presence of two OSNs^7^. In C type sensilla, three subtypes could be distinguished (Figs 1G–L and 2G–L). In *H. assulta*, the larger amplitude OSN was always tuned to Z9-16:Ald and Z9-14:Ald, while the smaller amplitude OSN responded to different stimuli in all three subtypes: Z9-14:Ald, Z9-16:OH, and Z11-16:OH in subtype C1; Z9-14:Ald and Z9-16:OH in subtype C2; only Z9-14:Ald in subtype C3 (Fig. 2G–L). In *H. armigera*, subtype C1 responded to four compounds: Z9-16:Ald, Z9-14:Ald, Z11-16:OH and Z11-16:Ac; subtype C2 to Z9-16:Ald, Z9-14:Ald and Z11-16:OH; subtype C3 to Z9-16:Ald and Z9-14:Ald (Fig. 1G–L).

**Figure 3:**
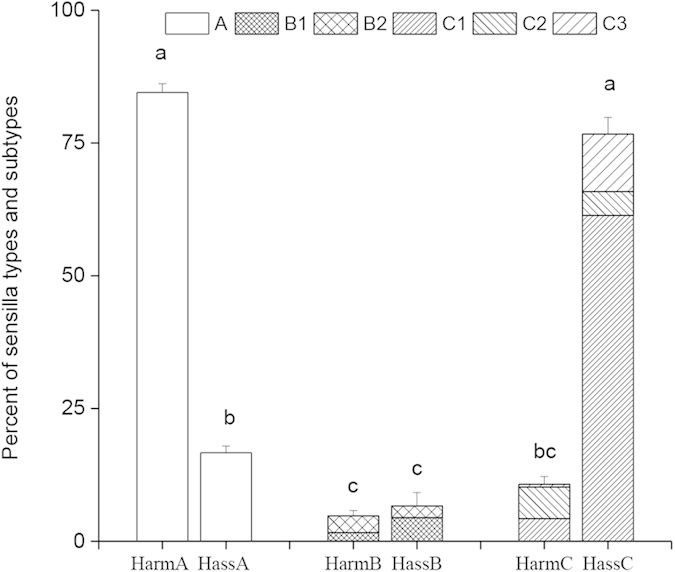
Relative abundance of type A, B and C sensilla and their subtypes in *H. armigera* (Harm) and *H. assulta* (Hass). The columns reporting abundance for sensilla of types B and C are divided into section to show the contributions of subtypes. For each species, three replications were conducted, and columns with different letters are significantly different at *P* < 0.05. Data are presented as mean + SEM.

The relative abundances of A, B1, B2, C1, C2, and C3 sensilla in the male antennae were found to be remarkably different in the two species (84.5%, 1.7%, 3.1%, 4.3%, 6.0%, 0.5% in *H. armigera*, and 16.7%, 4.4%, 2.2%, 61.4%, 4. 4%, 10.8% in *H. assulta*) (Fig. 3). In *H. armigera*, the type A sensilla dominate, being 7.9 times more abundant than those of type C. In *H. assulta*, instead, type C sensilla are most represented, 4.6 times those of type A. Type B sensilla are a minority in both species (Fig. 3). The dose-response curves of OSNs in the main types and subtypes of sensilla of the two species are shown in Fig. S1.

### Topographical expression pattern of PRs

**Figure 4:**
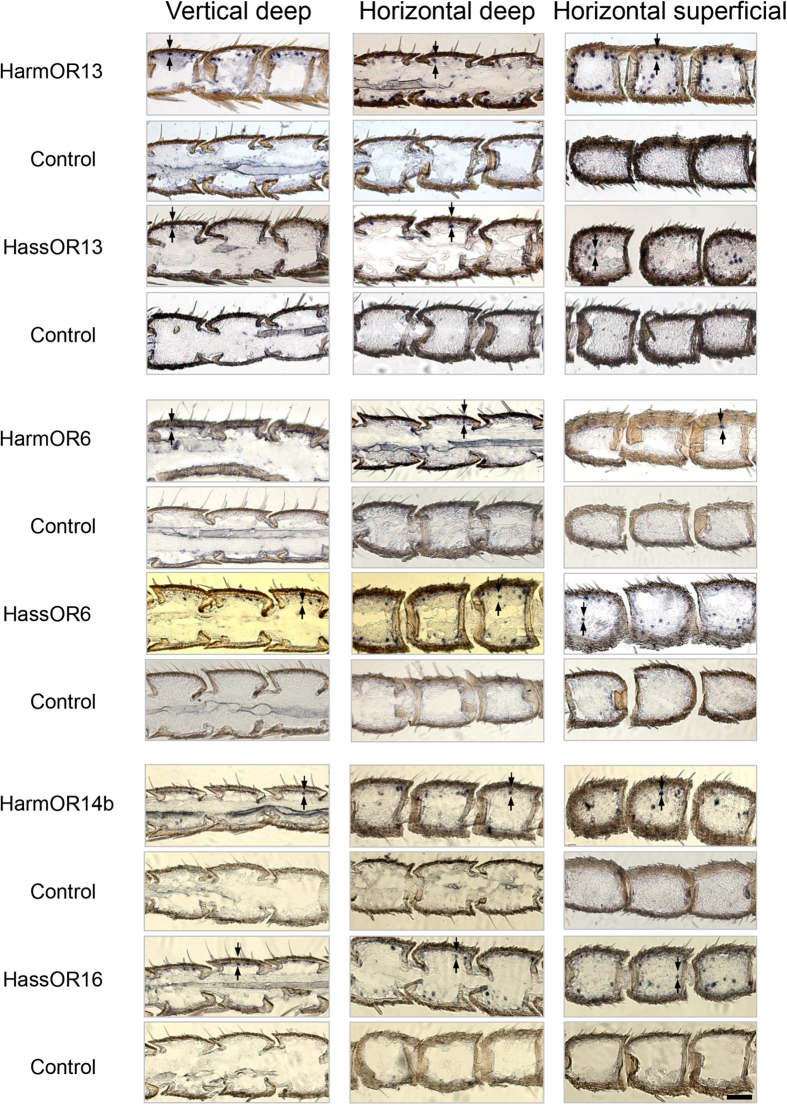
Expression patterns of selected ORs in *H. armigera* and *H. assulta*. Three sections (vertical deep, horizontal deep and horizontal superficial) were cut from male antennae and hybridized with antisense RNA probes for the indicated ORs, as described in the Materials and Methods section. The signals were visualized by Digoxigenin (DIG) antibody coupled with BCIP-NBT. The digoxigenin (DIG) labeled sense probe was used as the negative control. Arrows indicate the examples for stained cells. Scale bars: 50 μm.

**Figure 5:**
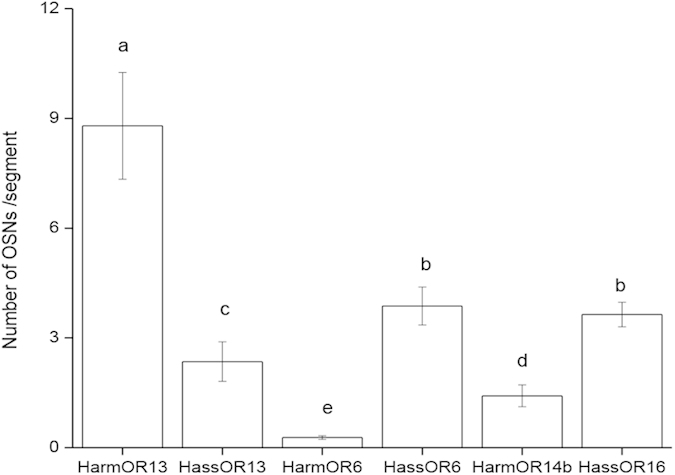
Numbers of OSNs expressing *HarmOR13*, *HassOR13*, *HarmOR6*, *HassOR6*, *HarmOR14b* and *HassOR16* in male antennae. Statistical analysis was based on the number of cell bodies of each OSN in all three kinds of sections (Fig. 4) of three antennae from three independent groups of experiments. Columns with different letters are significantly different at *P* < 0.05. Data are presented as mean ± SEM.

*In situ* hybridization results show the expression sites of HarmOR13 and HassOR13, HarmOR6 and HassOR6, as well as HarmOR14b and HassOR16 in the 30–60 annuli of male antennal flagella (Fig. 4). The signals were found to be annular, indicating that the riboprobes were hybridized with RNA that is perinuclearly distributed (Fig. 4). OR-positive cells were only detected on the side bearing sensilla, not on that covered with scale. The distribution of stained cells corresponds to the distribution of trichoid sensilla on the antennal surface. We selected antennae of three males for each species from three independent experimental batches to count the number of OR-positive cells from three kinds of sections. The average numbers of OR-positive cells from various annuli (varying from 3 to 9) of three kinds of sections on each antenna are graphically compared in Fig. 5. In the male antennae of *H. armigera,* the number of HarmOR13-expressing OSNs is 6.2 and 31.7 times higher than those of HarmOR14b and HarmOR6, respectively (Fig. 5). In the antennae of male *H. assulta,* the expression levels of HassOR6 and HassOR16 are similar, and about 1.6 times higher than that of HassOR13 (Fig. 5). When comparing the two species, HarmOR13-expressing OSNs in *H. armigera* are 3.7 times more abundant than those expressing HassOR13 in *H. assulta*. The ratio is reversed for the numbers of OSNs expressing OR6, 13.9 times higher in *H. assulta* than in *H. armigera*. Finally, the number of HassOR16-expressing OSNs in *H. assulta* is about 2.6 times higher than that of OSNs expressing HarmOR14b in *H. armigera*.

### Spatial representation of PGCs in MGC glomeruli

**Figure 6:**
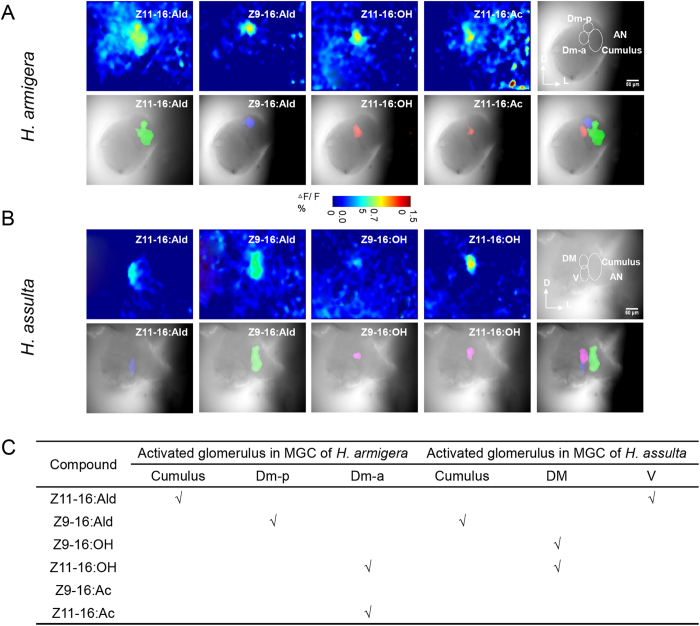
Patterns of responses to pheromone gland components in the antennal lobe (AL) of *H. armigera* and *H. assulta*. The antenna was stimulated with 100 μg of Z11-16:Ald, Z9-16:Ald, Z9-16:OH, Z11-16:OH, Z9-16:Ac, or Z11-16:Ac. (**A**,**B**) for each species, the upper row shows false-color-coded spatial response patterns of four active chemicals and the relative position of three glomeruli (the last image, D, dorsal; L, lateral); the lower row shows the corresponding activated patterns (exceeding 50% of maximum activity) superimposed on the grey-scale images of ALs and the activated glomeruli (the last image). (**C**): a summary of the activated glomeruli of the six chemicals.

Based on the AL atlas of *H. assulta* and *H. armigera*^27^,^30^, there are three MGC subunits in each species: cumulus, Dm-p, and Dm-a in *H. armigera*, and cumulus, DM and ventral unit in *H. assulta*. We identified the MGC subunits according to their positions and monitored their activities after stimulating the antenna with 100 μg of pheromone gland components (Fig. 6).

We had previously reported that Z11-16:Ald and Z9-16:Ald evoked responses in the cumulus and Dm-p in *H. armigera* respectively, while in *H. assulta* they elicited activities in the ventral unit and cumulus respectively^8^. Here we use the evoked area of these two components as landmarks within the MGC to determine the relative position of activities elicited by the other components. We found that Z11-16: OH and Z11-16: Ac both activated Dm-a in *H. armigera*, while Z9-16:OH and Z11-16:OH both evoked responses in DM in *H. assulta* (Fig. 6).

### Behavioral effects of PGC blends on male attraction in wind tunnel

**Figure 7:**
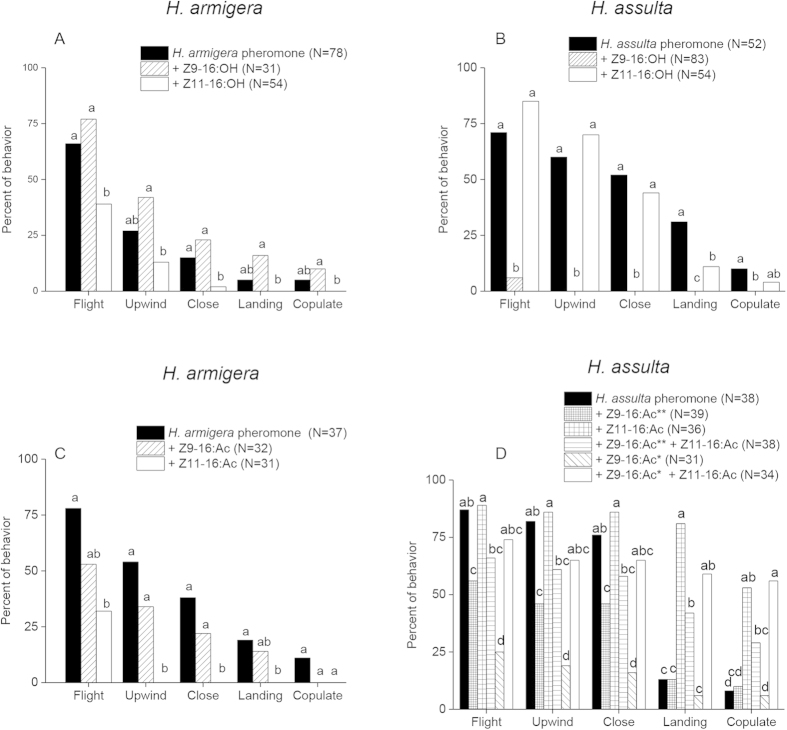
Behavioral responses of males of two *Helicoverpa* species to mixtures of pheromone gland components. To binary mixtures of Z11-16:Ald and Z9-16:Ald in the proportions found in the pheromone glands of each species, individual components were added, as indicated in the figure. Relative amounts of the components are reported in Table 2. In particular, panels (**A**,**B**) report the effect of added alcohols in *H. armigera* and *H. assulta*, while panels (**C**,**D**) show the effect of acetates. * indicates adding Z9-16:Ac in the amount of 15% of the basic pheromone blend; ** indicates adding Z9-16:Ac in the amount of 1.8 times that of the basic pheromone blend. Responses to the binary mixtures of aldehydes (black columns) were taken as references. Five behavioral effects were observed and quantified, as reported on the x-axes. Within each behavioral category, columns with no letters in common are significantly different at *P* < 0.05.

Based on the relative amounts of PGC in pheromone gland extracts of the two species, as previously studied in our laboratory^3^, ternary and quaternary blends were prepared by adding one or two components to the mixtures of Z9-16:Ald and Z11-16:Ald (Table 2), and the behavioral responses of males of the two species to each blend were studied in wind tunnel. (Fig. 7 and Table 2).

In *H. armigera*, addition of Z11-16:OH or Z11-16:Ac to the two-component blend inhibited a series of attractive steps during male pheromone-directed behavior, while Z9-16:OH and Z9-16:Ac had no significant effect (Fig. 7A,C).

In *H. assulta*, addition of Z11-16:Ac alone or together with Z9-16:Ac to the binary pheromone blend significantly increased males’ landing and displaying the copulation behavior (Fig. 7D). However, addition of Z9-16:OH or Z9-16:Ac significantly reduced the flight, upwind and close behaviors to the binary blend (Fig. 7B,D). Adding Z11-16:OH only reduced the landing behavior of *H. assulta* males (Fig. 7B).

## Discussion

Sex pheromone communication in moths allows males to find conspecific females over a long distance and also serves as a primary barrier of premating isolation among moth species^31^,^32^. Besides the principal pheromone components Z11-16:Ald and Z9-16:Ald, other chemicals in pheromone glands also play crucial roles in *H. armigera* and *H. assulta*. We comparatively study behavioral effects of PGCs and the olfactory coding mechanisms for PGCs in these two species, using state-of-the-art molecular, physiological and behavioral techniques to shed new light onto chemosensory bases of reproductive isolation between these species. Peripheral coding relative to these components in male is more complicated than previously suspected. Of the three types of pheromone sensitive sensilla (A, B and C) previously reported^10^,^11^,^13^, type B can be further classified into two subtypes and type C into three subtypes in both species. The abundance of OSNs expressing each of the 6 PRs in male antennae represents a guide to assign each PR to the OSNs in different types of sensilla. We found that Z11-16:OH and Z11-16:Ac act as antagonists for *H. armigera* in pheromone communication, while in *H. assulta* Z11-16:Ac is an agonist, and Z11-16:OH as well as Z9-16:Ac are antagonists. We also proved that Z9-16:OH acts as an antagonist for *H. assulta*^1^.

### Functional classification of the sensilla and their sensory neurons

The types of sensilla identified in *H. armigera* and *H. assulta* males seem similar to, but not as stereotypical as those described in several other Heliothine species before^10^,^11^. The A type sensilla always contain an OSN tuned to Z11-16:Ald, while the B and C type sensilla can be further classified into subtypes, B1, B2, C1, C2, and C3 (Figs 1 and 2). Such flexibility in the same types of sensilla implies that different groups of ORs could be expressed in the subtypes of B or C type sensilla. Sensilla of types B and C could be grouped together as both are mainly tuned to Z9-14:Ald, but may also respond to other chemicals. The presence of subtypes could be a phenomenon of chemosensory adaptation in speciation. The versatility of sensilla responding to a range of stimuli could increase the capability of males to detect other semiochemicals present in the environment, and may provide better adaptation to changes in pheromone components^33^. Such a divergence of chemical channels between the two sympatric species may be caused by either reinforcement or communication interference, but how changes in these communication systems evolve remains elusive^34^,^35^,^36^,^37^,^38^.

### Comparison of PR expression level and abundance of different types of sensilla

**Figure 8:**
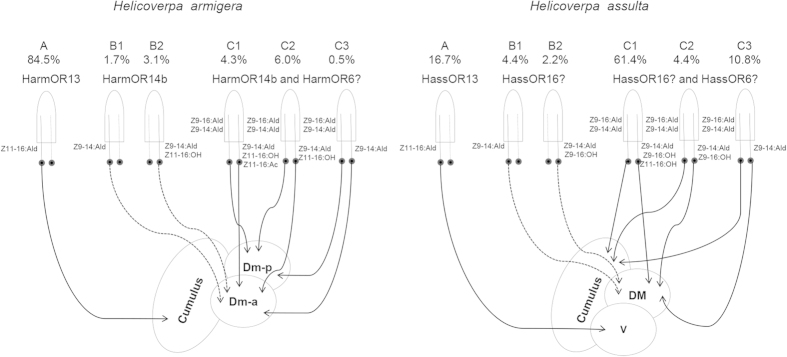
Model of peripheral coding of pheromone gland components in male antennae and antennal lobes of two *Helicoverpa* species based on the results of Wu *et al*.^6^ and the present study. The percentages of PRs in each sensillum type are based on the results of single sensillum recording and *in situ* hybridization (a question mark means uncertainty). Established (black lines) and speculated (dotted lines) connections between OSNs and dorsomedial unit; Dm-a, anterior dorsomedial unit; DM, dorsomedial unit; V, ventral unit.

The correlation analysis between the population sizes of the different types of sensilla and the numbers of OSNs expressing the associated ORs can help assigning different ORs to OSNs and types of sensilla in the olfactory system. HarmOR13 and HassOR13 when expressed ectopically in oocytes of *Xenopus* or in insect cells^24^, showed specific responses to Z11-16:Ald^18^,^20^. This compound is the major pheromone component of *H. armigera*, but is the minor pheromone component in *H. assulta.* The *in situ* hybridization results demonstrate that OR13-positive cells in the antenna of males of *H. armigera* are 3.7 times more abundant than in *H. assulta,* in agreement with the ratio of type A sensilla between the two species statistically (5.1, Fig. 8). Consequently, the OSNs expressing OR13 are definitely housed in the type A sensilla in both *H. armigera* and *H. assulta*.

Z9-16:Ald is the major pheromone component of *H. assulta*, and the C type sensilla in males of this species are predominant. However, the PR tuned to this chemical remains elusive. In our previous study, HassOR6 expressed in *Xenopus* oocytes exhibited a strong response to Z9-16:OH while displaying much smaller responses to Z9-16:Ald, Z9-14:Ald and Z9-16:Ac^18^. Xu *et al.* reported chemosensory receptor genes in *H. assulta* but failed to identify the PR responding to Z9-16:Ald^24^. More recently, Chang *et al.* reported HassOR6 as the PR tuned to Z9-16:Ald^14^. In the present study, we find that the HassOR6-positive cells as well as the HassOR16-positive cells significantly outnumber the OR13-positive cells in antennae of male *H. assulta*, but their population ratio of only 1.6 : 1, does not match the population ratio (4.6 : 1) between sensilla of types C and A in male *H. assulta* (Fig. 8). It is thus reasonable to suggest that HassOR6 and HassOR16 are expressed in OSNs of only some of the type C sensilla, and HassOR6 is not the PR tuned to the major pheromone component Z9-16:Ald in *H. assulta*.

HarmOR14b and HassOR16 have been found to be tuned to Z9-14:Ald^18^. We hypothesized that the number of HarmOR14b- and HassOR16-positive cells would be mirrored by the abundance of sensilla containing OSNs responsive to Z9-14:Ald, i.e. the sum of type B and C sensilla. The *in situ* hybridization results showed that the expression ratio of HarmOR13 to HarmOR14b in *H. armigera* is 6.2:1, in agreement with the ratio of type A to type B + C sensilla (5.5:1, Fig. 8). However, the ratio of HassOR13 to HassOR16 is 1:1.6 in *H. assulta*, much higher than the ratio of the related sensilla, 1:5.0 (Fig. 8). Thus, the expression level of HarmOR14b matches the abundance of OSNs tuned to Z9-14:Ald, i.e. a sum of type B and type C sensilla in *H. armigera*, but a similar model cannot be applied to HassOR16 in *H. assulta*.

To reliably characterize the different types of sensilla, the function and expression pattern of PRs they house need to be determined. Using specific riboprobes for each OR, *in situ* hybridization provides strong evidence to accurately define the topographical distribution of OSNs and sensillum types of interest in the antennae, especially for non-model animals like *Helicoverpa* species. Response profiles and distribution of OSNs in sensilla of different types (subtypes) provide the basis for further functional studies of ORs. It will be worthwhile to revisit the ligand profile of the various PRs, especially for OR6 and OR16 in this system.

### Behavioral valence of pheromone gland components

In our wind tunnel experiment, Z11-16:Ac was shown to act as an agonist for *H. assulta* males and as antagonist for *H. armigera* when added to the binary sex pheromone blends (Fig. 7D,C). Whether Z11-16:Ac works as a third sex pheromone component in *H. assulta* still requires further field tests. Our additional electroantennographic (EAG) experiments demonstrated that Z11-16:Ac is detected by the *H. assulta* antenna (Fig. S2). In ensuing SSR experiments we also found OSNs in several C1-type sensilla weakly responding to higher doses of Z11-16:Ac (Fig. S3). However, calcium imaging could not visualize representative areas of Z11-16:Ac activation in the MGC. Z11-16:Ac might not activate a single olfactory pathway, but may be involved in detecting the sex pheromones. In further studies we will add this compound to each sex pheromone component and their blends to see if it affects responses of the related OSNs and spatial representative patterns in ALs to sex pheromones.

Z11-16:OH and Z9-16:OH are common PGCs in pheromone gland extracts of Heliothine species^3^,^39^,^40^,^41^,^42^. Z11-16:OH is a minor sex pheromone component of *Heliothis subflexa*^43^ but an antagonist of many Heliothine species^2^,^44^,^45^. Our wind tunnel data indicate that Z11-16:OH and Z9-16:OH act as antagonists for males of *H. assulta*, in agreement with field data reported by Cork *et al.*^1^. Z11-16:OH is also proven to be an antagonist to male *H. armigera*. We find the OSNs in type C1 sensilla are tuned to Z9-16:OH and Z11-16:OH in *H. assulta*, which is different from the previous electrophysiological recordings done by Berg and Mustaparta^9^, in which no OSN responding to Z11-16:OH was found. Considering that the insect population in the previous study was from Korea, the difference might be related to this fact.

Cork *et al.* reported that addition of Z9-16:Ac and Z11-16:Ac to the main sex pheromone components decreased the trapped number of male *H. assulta* in China^1^. In our study, Z9-16:Ac is also found to be an antagonist. However, when both Z11-16:Ac and Z9-16:Ac were mixed with sex pheromone blends, the agonistic effect of Z11-16:Ac was so strong that it overshadowed the antagonistic effect of Z9-16:Ac (Fig. 7D). Our previous study showed that Z9-14:Ald acted as an agonist when minor amounts were added to the binary pheromone blends of *H. armigera*, but as an antagonist at higher levels. For *H. assulta*, Z9-14:Ald acted as an antagonist especially when presented with higher concentration^6^,^7^. These results indicate that these PGCs, Z9-16:OH, Z11-16:OH, Z9-16:Ac, Z11-16:Ac, and Z9-14:Ald also play roles in species isolation of related species of Heliothine moth.

In this study, we elucidate the behavioral valence of long overlooked additional sex pheromone gland components in the two species. Combining the results of the *in situ* hybridization, SSR, and calcium imaging in this study and from Wu *et al.*^7^, we can provide an overall picture of intra- and interspecific behavioral and olfactory responses to PGCs in *H. armigera* and *H. assulta*. A diagram summarizing the peripheral coding of PGCs in the two species is shown in Fig. 8. We identify different types and subtypes of sensilla containing OSNs responsive to PGCs and evaluate their abundance. We also confirm that HarmOR13 and HassOR13 are located in the type A sensilla and are involved in detecting Z11-16:Ald, a pheromone component of the main binary mixture in both species. Furthermore, based on the agreement between the ratios of sensillum numbers and OR abundance, we suggest that OR6 is just expressed in OSNs of some C type sensilla, while the PR tuned to the major pheromone component Z9-16:Ald in *H. assulta* need to be further identified.

## Materials and Methods

### Insects

*H. armigera* and *H. assulta* were originally collected as larvae in tobacco fields in Zhengzhou, Henan province of China, and successive generations were maintained in the laboratory under a 16 L: 8D photoperiod cycle at 26 ± 1 °C and 55–65% relative humidity. The larvae were reared on two kinds of artificial diets whose main component is wheat germ^46^. Pupae were sexed and males and females were put into separate cages for eclosion. After emergence, moths were fed with 10% honey in water. 2–4 days old virgin males were used in wind tunnel and *in situ* hybridization experiments. 2–6 days old virgin males were used for calcium imaging and electrophysiological studies.

### Chemicals

Odorants were selected for behavioral experiments and electrophysiology based on previously identified PGCs of female *H. assulta* and *H. armigera*^3^ 92% Z11-16:Ald, 90% Z9-16:Ald, 93% Z9-14:Ald, 92% Z9-16:OH, 92% Z11-16:OH, 90% Z9-16:Ac, and 92% Z11-16:Ac were purchased from Shin-Etsu Company (Tokyo, Japan). The purity of these compounds was increased to >98% by chromatography on a silica gel column. Solutions were prepared in HPLC-grade hexane or paraffin oil (Analytical grade, Fluka). All the solutions were stored in 2 mL glass vials (Agilent Technologies, Santa Clara CA, USA) at −20 °C.

### Abundance of antennal sensilla responding to PGCs

To classify the antennal sensilla along the male antennae of the two species and define the abundance of each type of sensilla, we used single sensillum recording (SSR) to measure responses of individual sensilla to seven PGCs, Z11-16:Ald, Z9-16:Ald, Z9-14:Ald, Z9-16:OH, Z11-16:OH, Z9-16:Ac, and Z11-16:Ac. In SSR experiments we used tungsten electrodes to record sensilla in the front of the antennae, and glass electrodes for the trichoid sensilla on the lateral sides of the antennae. We selected 140 sensilla from 23 antennae in *H. armigera* and 120 sensilla from 20 antennae in *H. assulta*, which were responsive to PGCs and located at 30–60 annuli of the antennae. In both cases, half of them were probed with glass electrodes and half with tungsten electrode. Three replications were performed for each species, and thus in total 420 sensilla in *H. armigera* and 360 sensilla in *H. assutla* were recorded. 100 μg (10 μL of 10 μg/μL solutions) of the seven compounds were used to determine sensilla types, while 100 ng, 1 μg, 10 μg, 100 μg, 1 mg were used for dose-response curves. Paraffin oil was adopted as control stimulus.

### Single sensillum recording (SSR)

The insect was placed inside a 1 mL disposable Eppendorf pipette tip with the narrow end cut to allow the head and the antenna to protrude. The head and antenna were immobilized with dental wax under a stereomicroscope. When recording with the tungsten electrode, the reference electrode was inserted into a compound eye, and the sharpened recording electrode was inserted into the base of a single sensillum in the front area of the antenna. When using the glass electrode for recording, sensilla on the lateral side of the antenna had their tips cut off using custom sharpened forceps, and inserted into glass capillaries filled with receptor lymph saline^47^. An Ag-AgCl electrode was placed in the glass micropipette to record action potentials of the receptor neurons. The recorded signals were then amplified through a IDAC interface amplifier (IDAC-4, Syntech, Germany). The software Autospike, version 3.4 (Syntech, Germany), was used to store and analyze data.

A continuous stream of purified and humidified air was directed on the antenna (12.5 mL/s) from the outlet of a steel tube (i.d. 6 mm, length 15 cm), positioned 2 cm from the antenna. Test odors were injected into the air stream using a stimulus flow controller (CS-55, Syntech, Germany), which generated 200 ms air pulses through the odor cartridge at a flow rate of 10 mL/s, and a compensating air flow was provided to keep a constant current. The odor cartridge was made with 10 μL stimulus loaded on a filter paper strip (0.7 cm × 2.5 cm) in a Pasteur pipette (15 cm long).

### Topographical expression pattern of PRs

To visualize the antennal expression sites of genes encoding HarmOR13 and HassOR13, HarmOR6 and HassOR6, and HarmOR14b, and HassOR16, we used *in situ* hybridization. We prepared slices of the 30–60 annuli of antennal flagella from the proximal end, making a vertical deep section, a horizontal deep section and a horizontal superficial section. Three replications were performed for each species.

### *In situ* hybridization

The protocol for *in situ* hybridization was adapted from Krieger *et al.*^48^. Briefly, fresh antennae were embedded in JUNG tissue freezing medium (Leica Microsystems, Germany). Sections of 12 μm were prepared with a Leica CM 1950 microtome at −22 °C, and, mounted on Superfrost Plus Glass Slides (Electron Microscope Science, Hatfield, USA). Slides were dried in air for 10 min, followed by fixing with 4% paraformaldehyde in 0.1 M NaHCO_3_, pH 9.5 at 4 °C for 30 min, then treated with phosphate buffer saline (PBS) (0.85% NaCl, 1.4 mM KH_2_PO4, 8 mM Na_2_HPO_4_, pH 7.1) for 1 min, followed by 0.2 M HCl for 10 min, and finally two washes with PBS for 30 sec. After rinsing in 50% deionized fomamide (MP Biomedicals, Solon, OH, USA)/5 × SSC (10 × SSC: 1.5 M NaCl, 0.15 M Na-citrate, pH 7.0) for 15 min, each slide was treated with 100 μL hybridization buffer (Boster, Wuhan, China) containing DIG-labeled probes. The slides were incubated at 55 °C for 14 h in a humid box wetted with 50% deionized formamide. Subsequently, the slides were washed twice with 0.1 × SSC for 30 min at 60 °C, rinsed briefly in TBS (100 mM Tris, pH 7.5, 150 mM NaCl), and incubated in 1% blocking reagent (Roche, Mannheim, Germany) in TBS plus 0.03% Triton-X100 (Merck, Darmstadt, Germany) for 30 min at room temperature. Then, each slide was incubated with 100 μL antidioxigenin alkaline phosphatase-conjugated antibody (Roche, Mannheim, Germany) diluted 1:500 in 1% blocking reagent in TBS plus 0.03% Triton-X100 at 37 °C for 1 h. After three washes for 5 min with TBS plus 0.05% Tween-20 and a brief rinse in DAP buffer (100 mM Tris, pH 9.5, 100 mM NaCl, 50 mM MgCl_2_), the signals were visualized by treatment with nitroblue tetrazolium (NBT)/5-bromo-4-chloro-3-indolyl phosphate (BCIP) (Promega, Madison, WI, USA). Digoxigenin (DIG) labeled sense probe was used as the negative control. Pictures were taken with a Leica DM 2500 microscope, and adjusted only for contrast and brightness by Adobe Photoshop CS3.

### RNA extraction and first strand cDNA synthesis

Antennae from twenty moths were homogenized in 1 mL Trizol (Invitrogen, Carlsbad, California, USA) and RNA was extracted following the manufacturer’s instructions. The RNA was treated with RQ1 DNase (Promega, Madison, USA) to avoid the risk of genomic DNA contamination, followed by purification with phenol, chloroform and isoamyl alcohol (Sangon, Shanghai, China). The quality of RNA was evaluated by agarose gel electrophoresis and its concentration measured with Nanodrop 2000 (NanoDrop Technologies, Wilmington, DE, USA). The first strand cDNA was synthesized from 2 μg RNA utilizing cDNA synthesis with SuperScript® III RT kit (Invitrogen).

### Synthesis of probes

Probes of different ORs were synthesized from linearized pGEM-T vectors containing the corresponding gene fragments cloned from cDNA of antennae of males of *H. armigera* and *H. assulta*. Digoxigenin (DIG)-labeled probes were synthesized with DIG RNA labeling kit version 12 (SP6/T7) (Roche, Mannheim, Germany). Biotin-labeled probes were synthesized by substitution of DIG RNA labeling mix with biotin RNA labeling mix (Roche, Mannheim, Germany). The probes were precipitated with 4 M LiCl and 100% ethanol, followed by a wash with 75% ethanol. The length of the probes were 1220 nt for HarmOR13 and HassOR13, 678 nt for HarmOR6 and HassOR6, 836 nt for HarmOR14b, and 668 nt for HassOR6. All the probes were fragmented into pieces of 300 nt by incubation with 80 mM NaHCO_3_, 120 mM Na_2_CO_3_, pH 10.2 at 60 °C. Probes were stored at −80 °C.

### Calcium imaging

The calcium imaging method was adopted from Galizia and Vetter^49^. Preparation of moths and optical recording were performed as described previously^7^,^8^. Briefly, virgin male moths were restrained in plastic tubes and fixed with dental wax. After dissecting and exposing the brain, we used a calcium-sensitive dye, CaGR-2-AM (Molecular Probes, Eugene, OR, USA) to stain the antennal lobes for one hour, and then thoroughly rinsed with Ringer solution. Imaging data were collected using a Till-Photonics imaging system (Till Photonics, Germany). The antennal lobe was illuminated at 475 nm. Stimulation started at frame 12 and lasted 500 ms in the recording sequence of 40 frames. For the false color images, the relative calcium change of each frame was calculated as relative changes in fluorescence (ΔF/F) by MATLAB software. 100 μg (10 μL of 10 μg/μL solutions) of six compounds (Z11-16:Ald, Z9-16:Ald, Z9-16:OH, Z11-16:OH, Z9-16:Ac, and Z11-16:Ac) was used as stimuli. Stimuli were applied in random order. Paraffin oil was used as control.

### Behavioral effects of PGCs

A wind tunnel was used to investigate the role of PGCs in the attraction behavior of *H. armigera* and *H. assulta*. A series of ternary and quaternary blends (Table 2) prepared with one or two more components added to the binary mixture (Z9-16:Ald and Z11-16:Ald) based on the PGC ratios^3^ were used as stimuli. Because Z9-16:Ac was found to be present at a quite high concentration in *H. assulta* female pheromone gland extracts (1.8 times that of the basic pheromone blend)^3^, we also used it at a lower dose (15% of the basic pheromone blend) according to concentrations identified in the related species *H. subflexa*^42^. The binary mixture was adopted as the control, and hexane was used as solvent. In each experiment, 10–20 male moths were tested and at least three replicates were performed. The order of treatments was randomized.

### Wind tunnel experiments

The males were tested during scotophase (4–6 hr) in a Plexiglas wind tunnel (2.5 m long, 1 m wide, 1 m high) at 24–26 °C, 40–60% relative humidity and 0.45 lux of red light. The wind speed was 0.5 m/s. Test samples were loaded on rubber septa and placed 30 cm above the floor at 40 cm distance from the upwind end of the tunnel. Males were acclimated in the wind tunnel room for 4 hours, then individually transferred to a cylindrical mesh cage (10 cm long, 5 cm diameter) and introduced into the downwind end of the wind tunnel at a distance of 180 cm from the odor sources, 30 cm above the floor. Five behavioral responses of males were categorized as follows: (1) Flight: male exited cage and flew toward test sample. (2) Upwind: male zig-zag or straightly flew at the height of sample source. (3) Close: upwind flight to within 10 cm of pheromone source. (4) Landing: male contacted the lure. (5) Copulate: male bent the abdomen and extended hairpencil to contact the lure. Every male moth was observed for 5 minutes.

### Data analyses

In SSR, spike frequencies (spikes/s) were calculated by counting the number of spikes during the first 200 ms of the response. For both non-responding and poorly responding OSNs, spikes were counted during the same 200 ms post-stimulus interval. Data of SSR were analyzed by the one-way ANOVA for analysis of variance, and the least significant difference (LSD) test was used for means multiple comparisons. To compare the number of olfactory receptor neurons on each antenna, neurons were counted from 3–9 flagellomeres. The number of olfactory receptor neurons between species, and between sections was compared by two-way ANOVA based on the Student-Newman-Keuls Tests. The ratios of different types of sensilla and OSNs expressing associated ORs were compared using Χ^2^ 2 × 2 test of independence with Yates’ continuity correction. In calcium imaging, data were acquired by the software Till-vision (Till photonics) and further analyzed by software ImageJ (NIH, USA) and custom-made programs in MATLAB (The Math Works, Inc). One-way ANOVA and LSD were used to compare the response intensity of different glomeruli in the MGC of tested compounds. In wind tunnel experiment, percentages of males in performing sequential behaviors were subjected to Χ^2^ 2 × 2 test of independence with Yates’ continuity correction. The level of significance was set as P < 0.05.

## Additional Information

**How to cite this article**: Xu, M. *et al.* Olfactory perception and behavioral effects of sex pheromone gland components in *Helicoverpa armigera *and *Helicoverpa assulta. Sci. Rep.* **6**, 22998; doi: 10.1038/srep22998 (2016).

## Supplementary Material

Supplementary Information

## Figures and Tables

**Table 1:** Pheromone gland components and their behavioral effects in *H. armigera* and *H. assulta.*

Pheromone gland components^3^	*H. armigera*	*H. assulta*	Behavioral effects
Z11-16:Ald	●	●	Pheromone component for both species^2^,^4^,^8^
Z9-16:Ald	●	●	Pheromone component for both species^1^,^8^
Z9-14:Ald	–	–	Agonist (0.3%)/antagonist (1% and above) for *H. armigera*^7^; antagonist for *H. assulta*^6^,^7^
Z7-16:Ald	●	•	No effects in *H. armigera*^2^ and no electrophysiology activity in *H. assulta*^9^
Z9-16:OH	•	●	Antagonist for *H. assulta*^1^
Z11-16:OH	●	●	Antagonist when mixed with Z9-16:OH for *H. assulta*^1^
Z9-16:Ac	–	●	Antagonist in China but agonist in Korea when mixed with Z11-16:Ac for *H. assulta*^1^
Z11-16:Ac	–	●	Antagonist in China but agonist in Korea when mixed with Z9-16:Ac for *H. assulta*^1^

Dot sizes represent the relative proportions of compounds in pheromone gland extracts; Dash means the compound was not detected^3^. Z11-16:Ald, (Z)-11-hexadecenal; Z9-16:Ald, (Z)-9-hexadecenal; Z9-14:Ald, (Z)-9-tetradecenal; Z7-16:Ald, (Z)-7-hexadecenal; Z9-16:OH, (Z)-9-hexadecenol; Z11-16:OH, (Z)-11-hexadecenol; Z9-16:Ac, (Z)-9-hexadecenyl acetate; Z11-16:Ac, (Z)-11-hexadecenyl acetate.

**Table 2:** The composition and ratio of pheromone blends used in the wind tunnel bioassays.

Pheromone blend^a^	Z9-16:Ald	Z11-16:Ald	Z9-16:Ac	Z11-16:Ac	Z9-16:OH	Z11-16:OH
Harm pheromone	2	98	0	0	0	0
Harm pheromone + Z9–16:OH	2	98	0	0	0.1	0
Harm pheromone + Z11–16:OH	2	98	0	0	0	6
Harm pheromone + Z9–16:Ac	2	98	15	0	0	0
Harm pheromone + Z11–16:Ac	2	98	0	11	0	0
Hass pheromone	95	5	0	0	0	0
Hass pheromone + Z9–16:OH	95	5	0	0	18	0
Hass pheromone + Z11–16:OH	95	5	0	0	0	2
Hass pheromone + Z9–16:Ac	95	5	15	0	0	0
Hass pheromone + Z9–16:Ac	95	5	181	0	0	0
Hass pheromone + Z11–16:Ac	95	5	0	11	0	0
Hass pheromone + Z9–16:Ac + Z11–16:Ac	95	5	15	11	0	0
Hass pheromone + Z9–16:Ac + Z11–16:Ac	95	5	181	11	0	0

^a^For making the pheromone blends, firstly each compound was dissolved in hexane with a concentration of 100 μg/μL, and then Z9-16:Ald and Z11-16:Ald were mixed in a total volume of 10 μL, and finally the third and/or fourth component was added according to the ratios.
