# Corrigendum: Olfactory perception and behavioral effects of sex pheromone gland components in *Helicoverpa armigera* and *Helicoverpa assulta*

**DOI:** 10.1038/srep24814

**Published:** 2016-04-29

**Authors:** Meng Xu, Hao Guo, Chao Hou, Han Wu, Ling-Qiao Huang, Chen-Zhu Wang

Scientific Reports 6: Article number: 2299810.1038/srep22998; Published online: 03152015; Updated: 04292016

This Article contains typographical errors in the Acknowledgements section.

“This work was supported by National Natural Science Foundation of China (grant number 31221091),”

should read:

“This work was supported by National Natural Science Foundation of China (grant number 31130050),”

