# Supplementary material for: Olfactory perception and behavioral effects of sex pheromone gland components in Helicoverpa armigera and Helicoverpa assulta
Source: Sci Rep. 2016 Mar 15;6:22998. doi: 10.1038/srep22998 (PMC4792173; doi:10.1038/srep22998)

Olfactory perception and behavioral effects of sex pheromone gland components in *Helicoverpa armigera* and *Helicoverpa assulta*

Meng Xu, Hao Guo, Chao Hou, Han Wu, Ling-Qiao Huang, Chen-Zhu Wang

Figure S1. Dose-response profiles of OSNs housed within type A, B, C1 and C2 sensilla in *H. armigera* male antennae; type A, B, C1 and C2 sensilla in *H. assulta* male antennae. Spike frequencies (spikes/s) were calculated by counting the actual number of spikes occurring during the first 200 ms of the response. Error bars report standard errors of the means. Spike frequencies are plotted against the amounts loaded on the filter paper.

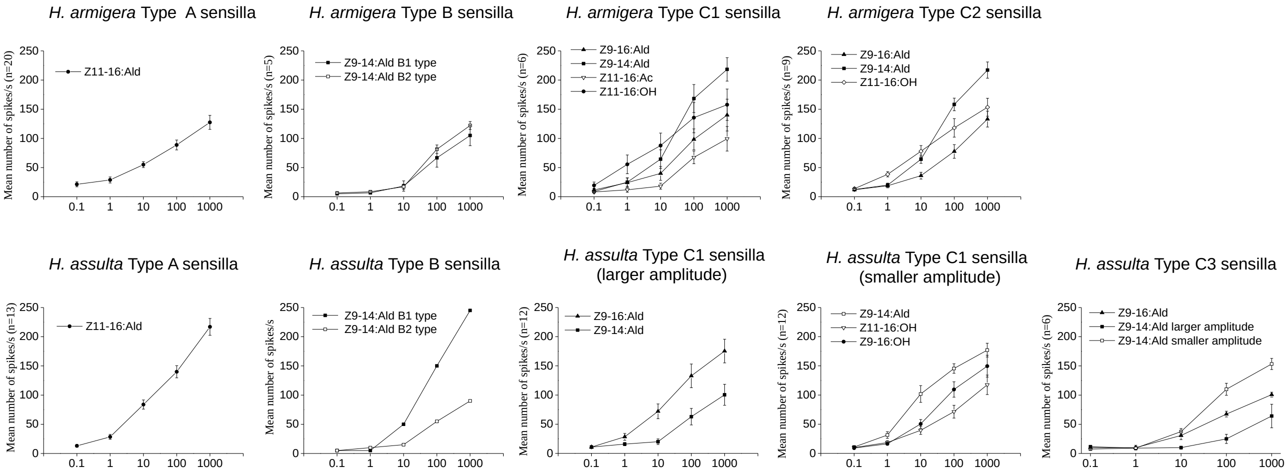

Figure S2. EAG responses to two acetate components of *H. armigera* and *H. assulta.* A: EAG responses of a male antenna of each species to Z9-16:Ac and Z11-16:Ac.B: Statistics on the results of EAG responses of male *H. armigera* and *H. assulta* to Z9-16:Ac and Z11-16:Ac. 10 µg (10 µL of 1 µg/µL solutions) of the two compounds were used. Hexane was used as control. Means in the same column followed by different letters are significantly different (One-Way ANOVA analysis, $P < 0.05$).

A

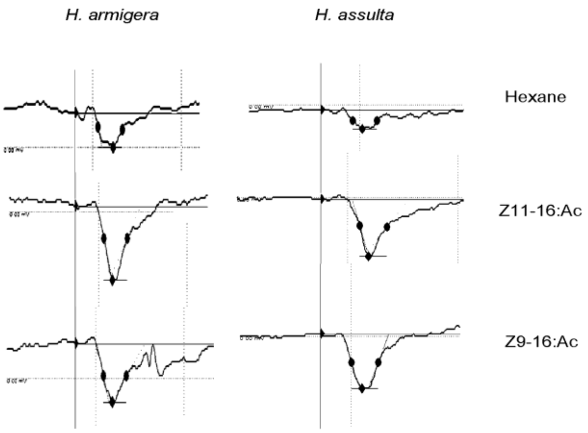

B

Compound	*H. armigera*		*H. assulta*	
	N	Mean±SE (mV)	N	Mean±SE (mV)
Hexane	16	0.293±0.038 b	22	0.285±0.016 b
Z9–16:Ac	16	0.518±0.063 a	22	0.443±0.029 a
Z11–16:Ac	16	0.566±0.067 a	22	0.436±0.022 a

Figure S3. Electrophysiological recordings of OSNs housed within one type C sensillum responding to Z9-16:Ac and Z11-16:Ac in *H. assulta.* The loading dosage of each compound was 100 μg except for the last two (10mg). The black bar at the top indicates duration of the stimulus. Paraffin oil was used as control.

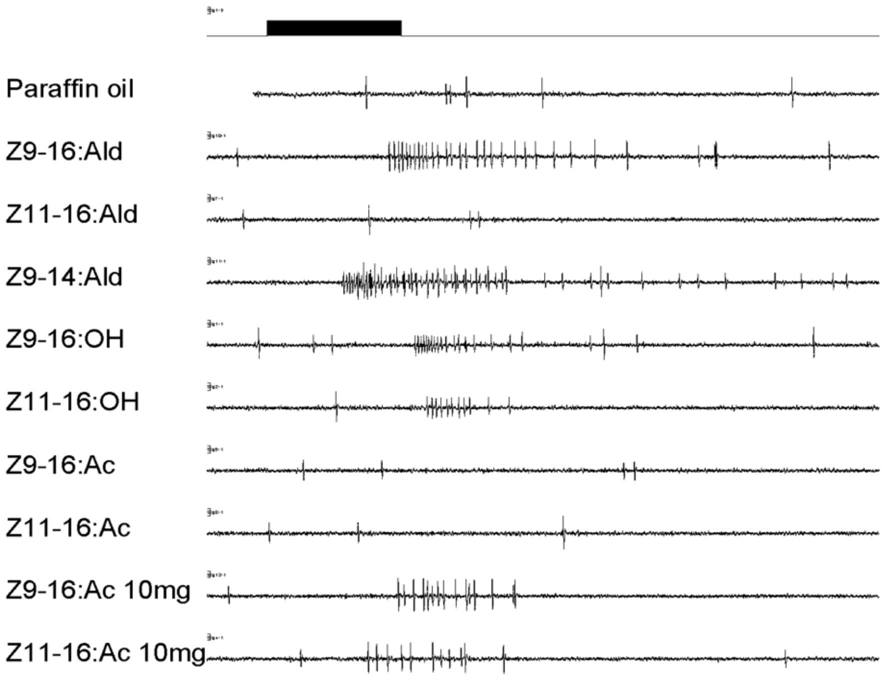

Supplement: Supplementary Information [file srep22998-s1.pdf]
